# Intermediary Perspectives on Total Worker Health in Small Businesses

**DOI:** 10.3390/ijerph181910398

**Published:** 2021-10-02

**Authors:** Thomas Cunningham, Brenda Jacklitsch, Reid Richards

**Affiliations:** 1 National Institute for Occupational Safety and Health, Cincinnati, OH 45226, USA; BJacklitsch@cdc.gov; 2 Centers for Disease Control and Prevention, Atlanta, GA 30329, USA; RRichards@cdc.gov

**Keywords:** small business, total worker health, safety and health

## Abstract

The future of work will include not only more small business employment, but also a need for greater consideration of more holistic approaches to addressing worker well-being. Previous research has suggested smaller firms need external assistance to add new or improve existing workplace health and safety activities. A Total Worker Health^®^ (TWH) approach is potentially appealing to small employers as it is intended to identify and support comprehensive practices and policies that take into account the work environment (both physical and organizational) while also addressing the personal health risks of individuals, thus being more effective in preventing disease and promoting health and safety than each approach taken separately. NIOSH researchers applied the NIOSH Small Business Intervention Diffusion Model to conduct parallel community-based TWH activities in two geographically distinct communities in a large metropolitan area. Data were collected from intermediary organizations that work with or serve small businesses about their perceptions of the TWH approach as a potential service for them to offer small firms. Intermediary organizations engaged in implementation of TWH approaches with small businesses in the respective geographic areas for approximately one year. Results indicated intermediary organizations find value in providing TWH assistance to small employers, but several challenges for intermediaries implementing TWH among small employers remain.

## 1. Introduction

The need to address both occupational and personal health in a more holistic manner has been called for to shape the future of work [1]. Studies have shown that workers with underlying health conditions may be at greater risk for certain occupational injuries and illnesses [2,3]. The consideration of both personal and occupational health and safety will continue to shape the future of work.

A significant challenge to addressing occupational safety and health (OSH) in general is that a large proportion of employers lack necessary resources to effectively manage OSH, as well as promote worker health and well-being. Most businesses are small—90% of firms have fewer than 20 employees [4], and small businesses are more likely to have limited resources to address health and safety [5]. As the business and workforce landscape evolves, average business size in terms of number of employees, is expected to continue on a downward trend [6]. Given the greater alignment of business and public health goals related to workforce health, and the limited resources to address workforce health management in the ever-increasing number of small businesses, the need for novel ways to deliver workplace safety and health assistance to small employers is as critical as ever.

The aim of this paper is to describe the application of the *NIOSH Small Business Intervention Diffusion Model* to better understand the experience of intermediary organizations in offering [5] and delivering assistance to small employers with implementing a Total Worker Health^®^ (TWH) approach, and thus help employers to address occupational and personal health, safety, and well-being more holistically. TWH is defined as “policies, programs, and practices that integrate protection from work-related safety and health hazards with promotion of injury and illness prevention efforts to advance worker well-being” [7].

Business size has been shown to be one of the best predictors of a small business’s involvement with workplace health promotion and safety activities such as TWH [8,9]. For businesses to survive, they need to sufficiently manage OSH [5,10], Promotion of occupational safety and health performance through programs such as TWH are critical to early business success. However, small businesses generally deliver *fewer* workplace health promotion and occupational safety and health activities than larger businesses [8,11,12]. In addition, small businesses endure a higher burden of occupational injuries and illnesses [13] and tend to need more external assistance with integrated safety and employee health programs such as TWH [14]. Furthermore, lack of resources, isolation, and inaccurate perceptions of illness and injury rates also contribute to small businesses’ lack of motivation to engage in prevention [9,15,16,17]. In 2012, only 34% percent of small businesses (defined as 50–100 employees in this example) offered even a minimal wellness program [18].

In spite of the limited ability to provide prevention-focused health and safety programs, the majority of small business decision-makers (93%) say the physical and mental health of their employees is important to their business’ bottom line [19]. Nearly half of small-businesses indicate that health and wellness programs are critical for recruiting and retaining the best employees, and nearly two-thirds believe such programs are worth the investment. The top three workplace health promotion (WHP) implementation motivators were identified as: (1) lower health care costs in the long term; (2) improved morale; and (3) increased productivity on the job [19]. Despite the value seen by employers, execution of such programs has been limited.

The use of intermediaries, or organizations that provide goods or services to small businesses, has been demonstrated to be an effective way of reaching small employers with health and safety assistance, and doing so through a trusted or at least a potentially acceptable source that a small businesses recipient might listen to [16,20,21,22,23,24]. Examples of intermediary organizations include, but are not limited to insurers, health providers, government agencies, suppliers, trade associations, and chambers of commerce. Such an approach requires that the intermediaries can be effectively engaged and acquainted with the public health source and the information to be communicated. For this study, the TWH concept and suggested programming were introduced to intermediary organizations and small businesses, with recognition of the need for multiple levels of intervention to support employee health promotion efforts, including support from the community [25,26,27] and working with intermediary organizations [9,21] in a small geographic area [28]. One assumption underlying this study was that development of a strong business case for TWH that included not only financial costs and benefits, but also personal effort required, time commitment, compatibility with existing systems, and expected outcomes, would be essential for participants to remain open to the concept and for the research team to potentially gain insight into successful future adoption [29,30].

The aims of the study reported here were (1) to understand perceptions of TWH among intermediary organizations that serve small businesses; and (2) to explore methods for encouraging promotion of TWH approaches by intermediaries to small businesses. In summary, this study attempted to address the question: How can the concept of Total Worker Health (TWH) be effectively introduced and delivered to intermediary organizations and small businesses using the NIOSH Small Business Diffusion Model? This report focuses on intermediaries’ experiences engaging in TWH-related outreach to small business employers in their respective communities. The goal is to shape the future of work by sharing key takeaways that may inform future TWH outreach and assistance efforts.

## 2. Materials and Methods

### 2.1. Procedure

**Figure 1:**
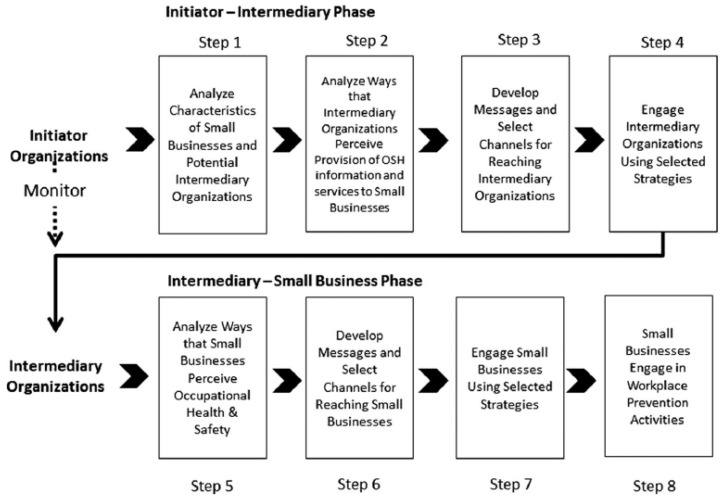
The intermediary–small business diffusion model.

The *Intermediary–Small Business Diffusion Model* was used as the basis for structuring and designing this study (Figure 1). The model was previously developed by the NIOSH Small Business Assistance Program, and its utility has since been demonstrated [22,23,24,31]. The model consists of two phases, an initiator–intermediary phase and an intermediary–small business phase, which provide a framework for identifying who small businesses use for information, how to reach out to those intermediaries, and how to work with those intermediaries to then reach out to and engage small businesses. This model is ideal for learning about both small businesses and intermediaries, as phases may overlap at multiple points. While the overall project applied both phases of the model, the results reported in this paper are focused primarily on the first, initiator-intermediary phase.

This part of the study included: (1) identifying and recruiting intermediaries, (2) deciding the best approach through discussions with the intermediaries, (3) intermediaries engaging with small businesses via consultations and offering TWH-related services, (which included a participatory research approach to having the intermediaries design and execute a pre- and post-interview data collection effort), and (4) asking intermediaries to reflect on the experience during a final debriefing focus group.

### 2.2. Identifying and Recruiting Intermediaries

This study targeted two communities in two geographically distinct, large communities in the same greater metropolitan area. These two communities were selected for convenience, as well as to explore perspectives of intermediaries in different types of communities (urban vs. suburban). Two sets of intermediaries were identified and recruited. The research team approached several intermediaries that were known to the research team through their memberships in local chambers of commerce. These intermediaries were considered likely to have some familiarity and experience in assisting employers with elements of a Total Worker Health approach, and included: occupational and business health services organizations, safety consultants, local health departments, and workers compensation insurers (see Table 1). Health departments were expected to be ideal partners because of their interest in new channels to deliver health objectives, experience in data collection, and interest in health outcome changes. Business health services were expected to be ideal partners because they already offered employee health services to businesses, were interested in market research, and understood the importance of both prevention and return-to-work activities. Safety consultants were expected to be ideal partners because of their knowledge related to workplace safety and regulations and their ability to discuss options to decrease risk of injury and illness.

### 2.3. Intermediary Discussions: Initial Perspectives on TWH

Data were collected during introductory meetings/discussions, which were key-informant interviews regarding intermediary perceptions about TWH. Meetings occurred 1–2 times for approximately 1 h with each participating intermediary. At the beginning of each meeting, the research team presented an overview of the TWH approach, which included the NIOSH definition and examples of TWH interventions [7]. The following responses were noted by a research assistant during the course of these introductory discussions with intermediary organization representatives (See Table 2). These questions and responses are included here to provide context for the experiences of the intermediary participants.

It should be noted that many of the wellness activities, especially through the health departments, would have been completed regardless of study participation, however additional funding was needed to provide the financial support for taking on and providing OSH assistance to the participating small businesses.

### 2.4. Intermediary Discussions: Formulating the Community-Based Approach 

Each set of community partners, or intermediaries, selected slightly different approaches for identifying small businesses as participants (see Table 3). Intermediaries were instructed that potential participating businesses were eligible if they had at least five but fewer than 50 employees [32]. Community A (an urban community) intermediaries selected a geographic approach which focused on 4 neighborhoods of previous interest, whereas Community B (a suburban community) intermediaries selected a sector-based approach (i.e., construction, childcare centers, manufacturing, municipalities). Community A included a focus on bringing businesses in the community together to pool funds for wellness and safety activities, while Community B tried collective activities in one sector (childcare) and engaged employers individually in the other sectors.

### 2.5. Intermediaries Engaging with Small Businesses Using TWH

Initial outreach efforts to small employers were conducted by the health departments via phone calls and interview data collection. Identifying local businesses already familiar to the participating intermediaries was helpful and contributed to the successful recruitment of 50 small businesses across the two communities from April 2014 to February 2015. Baseline interviews conducted by health department representatives with small business owner/managers in each community gathered initial perceptions about the TWH concept and determined what the business may be doing or may be interested in doing for employee personal health and occupational safety and health. While engagement with small employers was a part of the overall study, and preliminary results reported previously [33,34] indicated varying levels of successful engagement, the research question addressed within this paper is focused on the experience of intermediaries involved in offering TWH services to small employers.

The research team instructed the intermediaries to develop their own approach to offering TWH assistance to employers, with the general requirement that they offer services that collectively address worker safety, health, and well-being. One of the services that the intermediaries agreed to provide to the small businesses was a team consisting of a business health service provider and safety consultant, who would partner with the business to: (1) develop the mission and vision for the wellness and safety program, (2) develop goals and objectives for a customized wellness and safety plan, (3) implement the wellness and safety plan, (4) provide aggregate data report for screening (>20 participants), and (5) provide support in applying for safety and wellness grants.

While it was agreed that each employer would likely require a flexible, and customized approach, the intermediaries offered employers examples of services that could be provided during the course of the project, including: access to consultant expertise for wellness and safety for up to 3 onsite visits, as well as communication via phone or email; wellness/safety assessment; wellness/safety strategic plan; creative suggestions for enhancing wellness and safety programs and culture; participation in Employee Wellness Days (e.g., blood pressure screenings, health risk assessments, educational days about specific health topics). These service offerings were based on what the intermediaries suggested businesses are typically interested in, however changes to the service plan could still occur after individual business consultations. During these business consultations, consultants met with businesses and provided resources as requested.

Exit interview questions were collected by health department representatives in each community which were similar to those in the baseline interviews but also sought to elicit descriptions of changes in perceptions regarding TWH. Thus, all intermediary participants across the two communities were engaged with some TWH outreach and service delivery with small business employers during the course of the overall project.

### 2.6. Focus Group Interview Analysis

The final intermediary focus group interview was recorded and transcribed. The transcribed interview responses were analyzed by an inductive approach with thematic coding and a subsequent iterative process for further clarification of themes [35,36]. During familiarization, or phase 1, the research team independently reviewed the transcript and then together reached consensus on important themes and ideas. The team developed a codebook to guide data analysis. In phase 2 (generating initial codes), the research team systematically coded interesting features, direct quotations, and patterns across the entire interview, reviewing differences in coding until consensus was reached. During phase 3 (searching for themes), the data were reviewed, gathered into relevant groups, and collated into potential themes. Finally, the team conducted ongoing analysis to refine each theme and further condense the themes into a cohesive narrative. Text included in the results that appear in quotation marks are portions of the overall focus group transcript which are included to further demonstrate the participants’ experiences.

## 3. Results

The final intermediary focus group was composed of 11 attendees who work at health departments, hospitals, and other providers of safety or wellness/employee health services. The focus group began with asking about everyone’s overall thoughts on the concept of TWH, and then the discussion flowed from there to include participants’ perspectives on what worked well in implementing TWH, what did not work well, changes they have implemented based on their experience with the project, and ideas for how implement TWH approaches on a larger scale with small employers.

Overall, the intermediaries confirmed that small business employers care about their employees and are open and receptive to the idea of TWH and related new programming. They noted that wellness is a harder sell than workplace safety, and thus recommend that wellness be framed as a business proposition and emphasize the benefit to the company’s bottom line. Intermediaries reported that most small businesses already have safety programming which is often based on regulations in place, but wellness will have to be the add-on.

The intermediaries overwhelmingly confirmed the need for TWH programming that is flexible and tailored to the individual businesses. Intermediaries corroborated small business interviewees regarding the lack of resources or organizational structures to sustain TWH. Surprisingly, intermediaries reported that money was not a sufficient incentive to engage employers in health and safety programming. Instead, programs and initiatives that focused on bringing different small businesses together to pool resources and share events were more successful. Recognition and personal relationships were the primary motivators for small businesses when considering health and safety programming. Intermediaries suggested that the businesses needed to own the process for programs to be successful. They also mentioned that identifying one or two employees to be responsible points of contact and/or project coordinators was a factor for success.

The intermediaries from Community B that participated in the final focus group highlighted the childcare sector as the leader in TWH programming. They described specific TWH resources tailosector as “passionate” and very interested in TWH programming and described these experiences as the most successful.

### 3.1. General Reflections on the TWH Concept

Healthcare Provider A felt that that TWH made “a lot of sense”, and in their experiences, small businesses already had policies and communications prepared for safety, and they simply needed to broaden those messages to include wellness. They also mentioned a trend in the marketplace to include more on well-being, and how TWH fit into that as well. Safety Consultant B discussed the importance to trying to fit wellness in with safety and not the reverse, as many business, such as those in manufacturing, have a safety piece in place. One representative of Health Department A also mentioned the advantage of being able to get through the initial “door” by talking about safety and then say “Hey, by the way, let’s talk about wellness”.

A second representative of Health Department A described the TWH concept as innovative, as it had them thinking about safety and how it relates to residents in the community, such as how physical activity can be addressed from the safety perspective. Safety Consultant A also brought up that many small business employers are motivated by their own self-interest, and the need to stay open, paying employees. They also reported needing more from a risk management perspective, and that participating employers appeared “grateful for the assistance that they got with the recognition, evaluation, and control of hazards”. Safety Consultant A continued with describing TWH and this experience as entry point to the home, because “we’re interacting with people that spent a third of their lives, if they’re lucky, at work and why not that Total Worker Health^®^ and parts thereof be translated back home and underscored in a real public health perspective”?

### 3.2. Changes in Intermediaries Based on Experience with TWH Assistance for Small Employers

Some of the intermediaries shared that they changed how they talk to employers. Healthcare Provider A described how they were already heading down the “path of looking at people holistically and companies holistically” and that talking to people about their worksite culture of health and wellness provided insight into what drives the kind of support they have, what kind of communication they have, and what works from a communication perspective. They concluded by explaining that they had been previously asking about worksite wellness, but not safety; and that by learning about both, they had a “more rounded view of their worksite culture”. They also mentioned that they looks for opportunities now to include both safety and wellness, such as with respirator training and including discussion about tobacco cessation.

Safety Consultant B felt that with TWH, from the dollar and cents perspective, “we ought to be doing this”. They often work with businesses to address industrial hygiene/safety concerns, however, afterwards they ask the employer, “What support are you also offering your employees as far as wellness?” Even though this wellness piece may not be something offered, they want to draw attention to it since it keeps people healthier on the job, with less turnover, and fewer days away from work.

A couple of the intermediaries shared how the programming the offered to employers changed after their participation in the TWH activities. Health Department B described how they created a worksite wellness program for childcare providers that is now available on their state website. They went on to describe how this program has been shared by partners statewide.

Safety Consultant B described a situation where they asked workers what they hated most about their job, and were told about a tool that was difficult to use, “It was a pound a rod in the ground that deep and then there was a little teeny, wrench that you had to kind of twist at ankle height to get it out of the soil. Additionally, the people that were doing the job were, you know, it was people in their second-last career, you know, they’re overweight people with knees that don’t work but they had to do this”. Additionally, they asked the workers why they used the tool and was told that it came with the kit. They went on to describe how they found a fabricator to create a new tool so that the workers could stand up doing the task, making 300 of these tools per month, and back injuries have been reduced over this time with the new tool.

### 3.3. What Worked Well and What Didn’t Work Well to Assist Small Businesses with TWH 

Many of the intermediaries recognized that the small businesses had limited resources, and that communication was key to understanding the needs and limitations. One Health Department A representative described the need to help them understand the value and benefits of TWH. “You can meet the regulations that you need to while also sending your employees home at the end of the day healthier or better taken care of. I mean, having that communication was just- I cannot describe to you how important that was”. Safety Consultant A agreed, and went on to describe what they had experienced.

“They don’t have an organization structure that they can put this stuff into, they don’t have time, they don’t have money. What I found to be most attractive to the employers that we interacted with was almost a communal sense that really was palpable in [the neighborhood]. Where we were able to pull together different employers kind of through a loose-knit…business association. I think they’re generally aware of the demographics of their workers, they understand in some instances it’s an aging workforce. They’re concerned about turnover, but how do we pull these things together? In many instances they don’t even provide healthcare coverage. You really have to help them understand concepts like presenteeism, absenteeism, to help them get a sense for what’s going on. I didn’t get a sense that any of the employers needed an explanation from a return on investment perspective. They just needed to understand that if we can all come together, we can find the benefit”.

#### 3.3.1. The Value of Small Employer Groups

Another interesting aspect of the study that seemed to be positive was the formation of small groups of employers that participated in group discussions with the intermediaries. The employers could see the commonalities and bounce ideas off each other, and there was a positive peer pressure to participate. These employer groups also led to efficiency for some of the health services, as described by Hospital B: “…especially on the healthcare side we’re a little strapped for resources also, so, you know, if we can do things in large groups and be more efficient about things, that makes it easier for me”. Many of the intermediaries also noted that in the groups, the employers “learned and understood a lot more about health and safety because they started talking each other’s language. Additionally, before it was us trying to educate them on one-on-one when we first did the interviews about “This is what health is. This is what safety is”. However, when they heard their peers talking back and forth, I feel like they got a better understanding than us just talking to them”.

#### 3.3.2. Consistency of Engagement of Intermediaries with Small Employers

Intermediary opinions and experiences differed when it came to the consistency of engagement with the employers. For Health Department B, because they were already working with childcare centers, there was no handoff to another intermediary group after the recruitment period, and instead it was a smooth transition to TWH activities—this was seen as a factor that led to successes that were not necessarily seen in the other targeted industries (i.e., construction, manufacturing, municipalities).

Other intermediaries mentioned the lack of ownership or not having a single person be the key driver, resulted in difficulties. One representative of Health Department A explained, “Additionally, part of that comes with the fact that small businesses, you know, make connections with people, they are not large, corporate entities and they remember you—that you had this conversation with them and you built this relationship and so I had cultivated relationships and then I had to leave and then [another Health Department A employee] is trying to call and they’re like “Who’s [name of Health Department A employee 2]? Where’s [name of health department employee 1]”?

There were intermediaries that left jobs and came into new positions during the study which led to additional difficulties. Safety Consultant A described, “I know NIOSH is the umbrella, but I never felt like it was owned and it felt like we were in silos oftentimes where the communication was just- it was tough sometimes to try to figure it out [how] to get you guys and me and all you guys, all moving in one direction. … money is not a motivator [for small employers]. I think what motivated people was our listening- it’s person-to-person, you had the most success when you interact and they know who you are and that they can identify that’s something that you’re bringing”.

#### 3.3.3. Flexibility to Meet Employers’ Needs

Another key point that the intermediaries brought up was the need to have flexibility within the TWH program offerings to meet the employers’ needs. Many of the employer groups based in a neighborhood wanted to do things together, like lunch-and-learns, which seemed to give activities more “traction”. The intermediaries that worked with the childcare centers talked about how they first surveyed employees, and then held a health fair that included vaccinations and flu shots for all the childcare centers. Another intermediary mentioned that the pre-prepared model of activities they initially shared with the neighborhood group did not resonate, but once they allowed employers the flexibility to make changes that really made a difference.

### 3.4. Key Challenges to Implementation

Representatives from both health departments agreed that money was not a motivator for these businesses to try out TWH programming. They expressed surprise that the incentives to participate did not bring much success initially. Instead, they found that there needed to be an “energy around something” to motivate the businesses. A participant from Health Department A shared, “I think what worked more is when they were able to get together and they could bounce ideas off of one another. Additionally, then they started understanding together and then they started building once they understood together. That seemed to be the driver”. Additionally, Health Department B mentioned there were sector specific challenges. The example they provided expressed that the construction sector was particularly difficult to reach. They explained that, “You had to catch them when it was raining or when it was winter so that you could actually talk to them because as soon as it got sunny, they aren’t going to be in their office…”.

### 3.5. Organizations Best Suited for TWH Assistance for Small Employers

The intermediaries also shared their thoughts on what kinds of organizations might be best suited to assist with providing TWH to small businesses. One Health Department B representative shared that for some of the intermediaries, like the healthcare providers, “they’re in the business of business health and so we were partnering with them … trying to work under that umbrella, where they’re providing a service that is not taking away from their business, either”.

#### 3.5.1. Need to Take Advantage of Existing Networks, Not Create New Ones

Most representatives recognized the need to take advantage of existing networks, and not necessarily create new ones. Chambers of commerce were discussed as possibly being a helpful way to bring businesses together, however, it was also pointed out that the disadvantage with that organization is chambers charge a membership fee. Healthcare Provider A mentioned the advantage of neighborhood associations and good connections made through that option. Safety Consultant A pointed out that it was important to listen for and identify what resonates with the small businesses, “Am I hearing something from the women’s perspective? Is this a minority perspective? Where are the touchpoints for these people? Are they disabled veterans? These are all small business touchpoints that people will have in common and there’s not a lot of them but if you can have the good fortune to interact with them and have the ears to pick up on those kinds of issues or needs, you could put them together, you could make a bigger thing happen beyond just those handful of employers”.

#### 3.5.2. Small Versus Large Employers

The size and definition of small businesses were also discussed as a factor within the study and thinking about the future. Some, like Safety Consultant A, felt that increasing the number of employees in the small business definition, might have been better. Healthcare Provider A agreed, especially considering that in the near future, they would only be working with businesses with 100 or more employees. However, a representative of Health Department B mentioned that from their experiences, “small businesses are more like community than actual, larger businesses”.

### 3.6. Key Drivers to Make TWH Work for Small Businesses

The participating intermediaries had a few thoughts and ideas for what was needed to make TWH work for small businesses, including having more resources and intermediaries interested and available, clear messaging and examples of best practices, and the ability to reach new businesses.

Healthcare Provider A mentioned that they felt like more partners were needed that could provide free resources in order for TWH to be sustainable when there is not a large budget available for activities.

Health Department A mentioned that TWH was hard to “pin down” because “it’s everything”, so having key programs that worked in particular industries with data backing it up would help those starting activities at businesses in the future. Safety Consultant A agreed and mentioned that demonstrating success with outcomes and numbers was important. Promotion and messaging were mentioned by another representative of Health Department A as important, “We started to figure out a quicker way to say exactly what we were trying to do but I think in the beginning it was like two to three sentences and then we kind of got it down to a phrase and so I think messaging was important”.

A representative from Health Department A and a safety consultant from Community B both speculated that if possible, it would make sense to promote the idea of TWH early, to a business just starting out. They wondered how that might be introduced to a brand-new business in order to introduce and implement programming from the beginning.

## 4. Discussion

Given the rapidly evolving work environment where both occupational safety and health and personal health are considerations (which has been possibly amplified by the COVID-19 pandemic), and the continued shift toward more workers being employed by smaller firms [6,37], the need to understand effective methods for implementing TWH in small businesses is increasingly critical. The results of our community-based, participatory experience with several intermediary organizations suggest there is great potential for improving the dissemination and implementation of TWH approaches among the small business workforce.

There has also been increasing attention to understanding and overcoming OSH challenges among small business employers. Much of the most recent international small business OSH conference, the Understanding Small Enterprises Conference, included discussion of the changing nature and future of work [38]. Small business OSH issues continue to present myriad challenges, but several promising lines of research continue to drive knowledge about how to effectively improve OSH best practices in small businesses [39].

There have been several important efforts to address TWH among small employers. For example, Newman and colleagues have demonstrated an approach to reaching small employers with TWH-related assistance via a coordinating organization, Health Links ^TM^ [40]. This effort was launched with strong support from an insurance partner and a NIOSH Center of Excellence for TWH and has developed into a combination of a membership organization, credentialing or certification function, robust outreach and training delivery system, and networking opportunity for small employers based around TWH. Strong collaborative relationships are a key driver of its success and the results presented here suggest there is opportunity to involve many diverse intermediaries toward forming more customized and collective efforts around TWH among small businesses. Rohlman and colleagues have also demonstrated success in engaging small employers in both rural and urban environments [41]. In one example, the St. Louis Area Business Health Coalition has been serving as an intermediary organization delivering TWH to the St. Louis, MO business community [42]). As suggested by the health department representatives in this study, there may be opportunities to engage collectives of larger, more well-resourced employers and consider approaches to providing free and easily accessible assistance to smaller employers with interest to explore TWH approaches.

Identifying appropriate intermediaries for TWH efforts is also a consideration that needs to be well-thought out. As seen in this study, the health departments were great partners because they had: interest in finding new ways to reach out and deliver health messaging, experience in data collection, and interest in health outcome changes. Additionally, the business health service providers were good partners because: they already offer employee health services to businesses, they were not concerned with new clients but more interested in the market research aspect, and they understood the importance of both prevention and return to work activities. Additionally, of course, the safety consultants were ideal partners because of their expertise in workplace safety and regulations, and they could discuss options to decrease risk of injury at various businesses.

### Key Learnings

Several points can be taken away from the experience of intermediaries in delivering TWH assistance to small employers.

First, we may be overly reliant on our assumption that there is a need for significant financial support to enable greater engagement among intermediaries and small employers around TWH assistance and activities. At initial interview, most intermediaries expressed the assumption that financial resources would be among the most critical components to enable the planned TWH assistance activities with small employers. In the final intermediary focus group, there seemed to be agreement that motivation among employers, as well as social norms regarding responsibility for supporting health- and well-being-enhancing activities (versus pay), were key determinants to successful implementation of their planned activities for TWH assistance for small employers.

The intermediary participants in the baseline interviews and focus groups were aware of several prominent themes in their interactions with the small employer participants, which are reflected in the following summary of preliminary findings [33].

Overall, participating small employers have the motivation and willingness to incorporate safety and wellness into their workplace and recognize that they have not to date done enough. Participating employers have completed the requirements to meet OSH standards. They also have held small, informal activities to boost morale and support wellness. Despite their appreciation of safety and wellness as beneficial to their company’s success, many employers felt it was unlikely they would continue to offer wellness programming beyond their participation in the study primarily because of lack of budget, time, and easily accessible resources. After money, the other most salient challenges for employers trying to engage in new TWH program elements are time, employee interest, and a mindset that health and safety should be driven by employees’ individual responsibility with the site offering support and resources as feasible. At the start of the study, respondents almost universally discussed safety and wellness as separate issues. When asked about integrating, some believed it made sense to integrate wellness and safety as part of the same overarching program. However, about half of the respondents felt that safety should be kept separate. Because of the broad diversity of employees, size of company, type of work, and environmental context, a “one-size-fits-all” integrated TWH program would likely not work across participating sites. Although cost, time, and employee interest were overarching challenges, respondents described assets and challenges unique to their workplace that would be crucial to consider in designing a successful program.

Based on the perspectives of the participating intermediaries, it is evident that a cultural shift will be necessary to implement TWH programming. As the intermediaries observed in their interactions with small employers, respondents expressed interest and willingness to support employee health/wellness and safety, but there was not the sense that they saw wellness as a fundamental part of their workplace. This may further support the suggestion that a multi-layered approach to supporting small employers in addressing worker safety, health and well-being is needed from intermediary organizations. Not only do many employers not see themselves as fundamentally responsible for personal health and well-being of individuals they employ, they also lack adequate resources to adopt and manage new approaches and activities.

It has been suggested that key drivers of small firm culture include the owner/manager, as well as the community with which the firm identifies [5]. The results presented here compare and contrast a geographically defined and a sector defined community approach to engaging small employers in TWH activities. There may be positive and negative aspects of both approaches, and the experience of the intermediaries described here suggests this is perhaps a key element of a customized approach to disseminating and implementing TWH approaches among small employers.

Perhaps the most challenging step in initiating the TWH diffusion demonstration described here is identifying and engaging with potential champions and supportive opinion leaders within intermediary organizations. The work described here has followed an ongoing OSH research and outreach effort which has included active participation by the research team in various business networks, associations, and other organizations that either do, or have potential to offer, OSH assistance to small employers. Potential intermediaries must have top level support, individual interest that adds to their motivation, and a desire to work with small businesses, whether driven by goals related to finding new clients, adding members, reaching more service recipients, or other organizational objectives. Each of the intermediaries approached for this project were known to have some ongoing workplace health and/or safety role, but not all were necessarily receptive to the concept of TWH. These challenges highlight some weaknesses of the intermediary model, including turnover of champions within intermediary organizations, shifting priorities, and inconsistent funding support.

As suggested by the Small Business diffusion model [22,23], it is key for the initiator of TWH diffusion efforts to monitor activities of intermediaries, and take an active and participatory role in the community of interest. There is a delicate balance between sharing knowledge with intermediary partners and allowing them to act upon the knowledge in a way that will be sustainable after the initial demonstration of the initiator-intermediary-small business relationship. There is also an important social exchange occurring in each instance of communication between the initiator (here, the research team) and intermediary participants when sharing TWH knowledge. That is, the intermediaries must perceive value in the exchange, and thus act upon the knowledge, in a way that is compatible with their organizational practices, policies, culture, etc. The research team in this project aimed to share the concept of TWH as well as the importance of assisting small employers clearly with the intermediary partners, and then observe and capture a record of their experience in engaging small employers around the concept of TWH. For example, a safety consultant partner was lost due to lack of top-level support for providing workplace health promotion assistance. The intermediary organizations developed their own approach to engaging small employers, and they also observed what worked and what did not in delivering TWH services to small businesses.

Based on both our results presented here and previous work in reaching small employers with TWH approaches, flexibility is key. TWH looks different in each organization and likely varies in program elements included, leadership of TWH activities, and evaluation of outcomes (both perceived and measured).

The way TWH is described by intermediaries may not align directly with the prescribed definition (NIOSH, 2017), and part of the adoption of TWH among intermediaries and small employers may veer between more academically precise and business/organizational terminology. For example, organizations engaged in this project frequently used the terms “workplace safety and wellness” instead of “health promotion and occupational safety and health”. Although the definition of TWH was clearly shared and understood among the intermediaries, much of the practical discussion among intermediaries and small employers included reference to ‘wellness’, which is clearly not synonymous with TWH (NIOSH, 2021).

Incentives may be needed to motivate intermediaries to assist small businesses. While monetary support seemed be less of a concern in interactions between intermediaries and small employers, the intermediaries involved in this project all received some level of financial support to engage in this project, and in some instances these scarce public health/OSH research financial resources were noted as a limiting factor among the intermediaries. Some financial assistance for small employers to add new TWH-related elements to their safety and health management systems has been shown to be effective in increasing uptake of TWH interventions (e.g., grants from workers’ compensation insurers [43]; Health Links business grants [40]). While preliminary results suggested a $50 participation incentive may be insufficient to entice employers to engage in opening discussions of TWH [33], exit focus group results suggest financial incentives were not a strong motivator for employers to engage with intermediaries on TWH. Clearly there is ongoing need for use of scarce health and well-being resources to be creatively and efficiently applied to assist small employers, which will likely be in greater demand as future workplaces will need to address worker well-being to remain competitive and survive.

Evaluation and feedback are critical in launching a new collaborative effort such as those described here, and are also a part of the social exchange between the initiator and intermediaries which has been noted as adding value for intermediaries [23]. In this project, it is difficult to assess any quantifiable effect ongoing feedback from the research team had on intermediaries’ increased adoption of TWH, however, several instances of intermediaries disseminating results of their experiences with engaging small employers in TWH to their own peer networks (e.g., presentations by intermediary participants to national and state professional conferences) suggest there was substantial value perceived by those participating intermediaries.

## 5. Conclusions

This study explored how the concept of Total Worker Health can be effectively introduced to community organizations, so that they may in turn deliver TWH assistance services to small businesses.

It has been known that limited resources are a central barrier to implementing safety and health interventions. This study adds to the limited, but growing literature that suggests a need for financial and programmatic support for intermediary organizations able to assist small employers with implementation of TWH approaches to managing worker well-being.

Interestingly, the COVID-19 pandemic has synergized many business and public health interests, as evidenced by employers’ efforts to follow public health recommendations to limit the spread of SARS-CoV-2, encourage employee vaccinations, and in some cases, bolster trust in public health [44]. Although this study pre-dates the COVID-19 pandemic, the findings from discussions with intermediaries seem more relevant than ever as employers struggle with meeting greater demand for OSH management while also facing business survival challenges as the nature of work continues to evolve, perhaps in even more rapid and unanticipated ways.

## Figures and Tables

**Table 1:** Intermediaries for two communities.

Community A	Community B
Health Department A	Health Department B
Healthcare Provider A (Hospital Wellness Services)	Healthcare Provider B (Hospital Healthcare/Business Health Services; includes ergonomist)
Retired Workers Compensation Safety Consultant	Workers Compensation Provider
	Safety Consultant

**Table 2:** Questions and representative responses with intermediary organization representatives.

Question	Response
What is your reaction to TWH (as described with the NIOSH definition and examples of TWH interventions [7])?	Healthcare Provider B stated they thought about TWH in terms of preventive health maintenance and post-injury care. Health Departments (A, B) reported limited focus on OSH. Instead, their engagement with employers was mainly focused on workplace health promotion or wellness, with emphasis on physical activity and smoking cessation activities.
How compatible is the “TWH for small businesses idea” with the past experiences, existing range of program products, and strategic directions of this organization?	Healthcare Provider B stated they felt TWH was fairly compatible because they already had some activities related to wellness and safety, which were similar to the examples of TWH interventions described by the research team (e.g., ergonomics consultations with employers). Health Department A felt they did not have any programs currently related to OSH. Instead, their work–life programs tended to focus on wellness.
How difficult would it be for your organization to try delivering TWH ideas and services to small businesses?	Health Department A noted a barrier for their organzation, specifically, people are unlikely to perform a task not in their job responsibility. Healthcare Provider B noted low number of employees per business, and therefore business may not be able to afford services.
Compared to other business improvement ideas that your organization offers to small businesses, how difficult is the TWH idea to understand for people in your organization?	Healthcare Provider B speculated that some might think of “at work” vs. “outside of work” while others might think of preventive health maintenance versus post-injury care.
Would a subsidy make a difference in the willingness of your organization to offer TWH information and/or services to small businesses? If so, how would different subsidy levels make a difference?	The overall opinion was that money makes a difference in most cases: Healthcare Provider B felt offering each business some TWH activities about 4 times per year seemed doable for roughly USD 20,000. In regard to worker safety and health and tax credits or incentives, Health Department A noted that when small businesses have an opportunity to receive a financial reward, the option for financial incentives becomes the benefit most preferred by small businesses employers. Money was not a factor with the Workers’ Compensation Provider in Community B, because they were simply not interested in wellness. [This group eventually withdrew from participating as a potential intermediary.]

**Table 3:** Eligibility and outreach strategies for two communities.

	Community A	Community B
Targeted businesses	Located within 4 neighborhoods	Sectors: manufacturing, construction, childcare, municipalities
Business Size	5 < eligible < 50 employees	
Outreach	Phone calls or walking the neighborhood and dropping in to distribute information	Phone calls

## Data Availability

The data presented in this study are available on request from the corresponding author. The data are not publicly available due to privacy concerns.
